# Effect of Beta-Carotene Supplementation on the Serum Oxidative Stress Biomarker and Antibody Titer against Live Bovine Respiratory Syncytial Virus Vaccination in Japanese Black Calves

**DOI:** 10.3390/vetsci5040102

**Published:** 2018-12-14

**Authors:** Konosuke Otomaru, Rei Ogawa, Shoko Oishi, Yuki Iwamoto, Hyeyoung Hong, Kathuhisa Nagai, Koji Hyakutake, Chikara Kubota, Takahiro Kaneshige

**Affiliations:** 1 Joint Faculty of Veterinary Medicine, Kagoshima University, Kagoshima 890-0065, Japan; ogawa_000@outlook.com (R.O.); k7919886@kadai.jp (S.O.); yuki00ls601@yahoo.co.jp (Y.I.); v.forsythia@gmail.com (H.H.); katsuhisa-n@vet.kagoshima-u.ac.jp (K.N.); k3219851@kadai.jp (K.H.); k7980336@kadai.jp (C.K.); 2 Department of Veterinary Medicine, Kyoto Biken Laboratories, Inc., Kyoto 611-0041, Japan; kaneshige@kyotobiken.co.jp

**Keywords:** antibody titer, beta-carotene, bovine respiratory syncytial virus, Japanese Black calf, oxidative stress, vaccination

## Abstract

The purpose of this study was to determine the effect of vaccination and beta-carotene supplementation on blood oxidative stress and antibody response in calves. Thirty Japanese Black calves were randomly assigned to two groups. Fifteen calves received 20 mg of beta-carotene supplemented into their daily provided rations from 2 to 8 weeks of age (BC group), and the other 15 calves did not receive the daily beta-carotene supplement (control group). All calves received a commercially available modified live bovine respiratory syncytial (RS) virus vaccine at 4 and 8 weeks of age. Blood samples were taken at 2, 4, 8, and 12 weeks of age. At 4 weeks of age, the concentration of reactive oxygen metabolites within serum were significantly lower in the BC group than the concentrations measured in the control group. Also at 4 weeks of age, the concentration of biological antioxidant capacity within serum was significantly higher in the BC group than the concentrations measured in the control group. Both groups showed a gradual decrease of antibody titers to live bovine RS virus in the samples taken from 2 to 12 weeks of age. These results confirmed that beta-carotene supplementation decreased oxidative stress. However, beta-carotene supplementation did not affect the antibody response to live bovine RS virus vaccination, perhaps due to the presence of the maternal antibody.

## 1. Introduction

Young suckling calves, when compared to cows, have immature immune systems as evidenced by their lower numbers of lymphocytes in the peripheral blood, which are responsible for humoral and cellular immunity [1]. This was demonstrated by fairly low antibody response to vaccination [2]. Bovine respiratory syncytial (RS) virus causes bovine respiratory disease, which results in severe symptoms with concomitant bacterial infections [3]. To prevent infection of the bovine RS virus in calves, vaccination with the modified live bovine RS virus is occurring in Japan.

Beta-carotene plays an important role in the function of the immune system and also functions as an antioxidant inside the body for maintaining the stability of biological membranes [4,5]. Many studies of human and animal supplementation with beta-carotene have reported improvements in efficient quenching of singlet oxygen and immune function [6,7,8,9,10]. Oral supplementation of beta-carotene in humans resulted in decreased production of blood oxidative stress and increased antibody response to vaccination [9,10].

Therefore, supplementation of beta-carotene to Japanese Black calves was expected to decrease blood oxidative stress and improve antibody response to vaccination. There has been increased interest in determining the mechanisms by which oxidative stress influences metabolism and health in animals [11]. The measurement of endogenous hydroperoxide, as derivatives of reactive oxygen metabolites (d-ROMs), is a simple, reliable, and inexpensive method with high accuracy and linearity [1]. Hydroperoxide is a suitable biomarker for assessing oxidative stress [12]. The biological antioxidant capacity (reducing iron from its ferric (Fe^3+^) form to ferrous (Fe^2+^) form in serum samples) is measured by a biological antioxidant potential (BAP) test. The BAP test provides a global measurement of many antioxidants [13]. Although several studies have reported antibody response to vaccination and degree of oxidative stress in calves [14,15], there has been no report on the relationship between supplementation of beta-carotene and blood oxidative stress, or between supplementation of beta-carotene and antibody response to vaccination in calves. Additionally, the metabolism and meat quality of Japanese Black cattle are different from other cattle breeds, and Japanese Black calves tend to be susceptible to diseases such as diarrhea [16,17]. Furthermore, the value of Japanese Black cattle is very high and the price per head has been increasing in recent years.

The purpose of this study was to evaluate the effect of beta-carotene supplementation on the serum oxidative stress biomarkers and the antibody response to modified live bovine RS virus vaccination in Japanese Black calves in order to keep calves healthier.

## 2. Material and Methods

### 2.1. Study Design

In this study, we used 30 Japanese Black calves kept at one farm in Kagoshima Prefecture, Japan. All samples were collected from November 2017 to May 2018. All the calves stayed with their mothers for 4 days after birth and were housed indoors. Starting at 5 days after birth, they were fed with milk replacer and were raised individually in calf hutches. The amount and nutrient composition of feed are shown in Table 1. The calves were randomly assigned to two groups. Fifteen calves were orally supplemented with 20 mg (the dose was based on the study by Bierer et al. [18]) of beta-carotene (Rovimix beta-carotene, DSM Nutrition Products, Basal, Switzerland) once daily from 2 to 8 weeks of age (BC group), and 15 calves were not supplemented with beta-carotene (control group). All calves were managed in the same manner and fed to meet their nutritional requirements according to the Japanese beef cattle feeding standard [19]. All calves were vaccinated with modified live bovine RS virus vaccine (No. 52 strain, Kyoto Biken Laboratories Inc., Kyoto, Japan) at 4 and 8 weeks of age following manufacturer’s instruction. Blood samples were taken at 2, 4, 8, and 12 weeks of age from the jugular vein using Vacutainer plain tubes. Serum was isolated from the blood samples by centrifugation and stored at −30 °C until analysis. No calves developed disease during the study period. The calves were raised according to guidelines of animal care of the Joint Faculty of Veterinary Medicine at Kagoshima University.

### 2.2. Measurements and Analysis

The serum beta-carotene and zinc concentrations were measured using the 7020 clinical autoanalyzer (Hitachi High-Technologies, Tokyo, Japan). The measurement of the serum beta-carotene concentration was carried out by a colorimetric method. The beta-carotene measuring reagents have been commercially available since 2012 and have been widely used in Japan (beta-carotene measuring reagents, Kanto chemical, Tokyo, Japan). Two reagents were used for the measurement of beta-carotene. Initially, bilirubin in the sample was quenched by the oxidizing agent in reagent 1. Next, the beta-carotene in the sample was quenched by reagent 2. Changes in absorbance were calculated as the concentration of beta-carotene. The patent number of the reagent in Japan is 056086867. The measurement of serum zinc concentration was also performed by a colorimetric method using a commercially available zinc measuring reagent (Acurus auto Zn, Shino-Test, Tokyo, Japan) [20]. Serum concentrations of retinol was determined using high-performance liquid chromatography (Prominence, Shimazu, Kyoto, Japan) [21]. The serum antioxidant capacity was determined as the concentration of BAP, and the serum free radicals were determined as the concentration of d-ROMs. The serum BAP and d-ROMs were measured using a free radical analyzer (FREE carrio duo, Wismerll, Ltd., Tokyo, Japan) [11,12]. The serum d-ROMs values were expressed as arbitrary “Carratelli units” (Carr U). 1 Carr U corresponds to 0.8 mg/L hydrogen peroxide. The serum antibody titers to bovine RS virus were determined by a neutralization test. The neutralization test was performed as previously described by Kubota et al. [22].

### 2.3. Statistical Analysis

Data of the serum zinc, beta-carotene, retinol, BAP, and d-ROMs are expressed as the mean ± standard deviation. The serum antibody titers to bovine RS virus are expressed as the geometric mean ± standard deviation. The statistical analysis was conducted to determine the differences between the two groups within the same weeks of age, as well as between weeks of age within the same group using Student’s *t*-test with SPSS statistics 24 software (IBM, Tokyo, Japan).

## 3. Results

**Figure 1:**
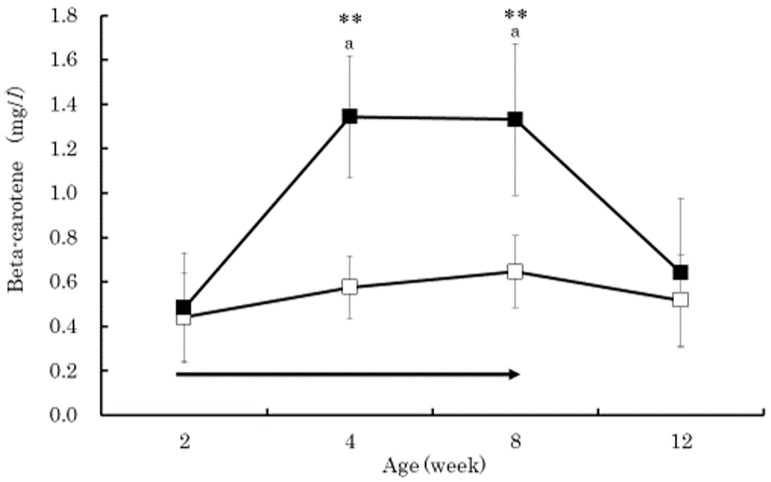
Changes in serum concentrations of beta-carotene in the BC group (dark square) and control group (empty square). Data are shown as mean ± SD. Arrow indicates beta-carotene supplementation period. Asterisks indicate a significant difference between groups at the same age (**: *p* < 0.01). Same lower case letters (a) indicate a significant difference from 2 weeks of age within the BC group (*p* < 0.01).

**Figure 2:**
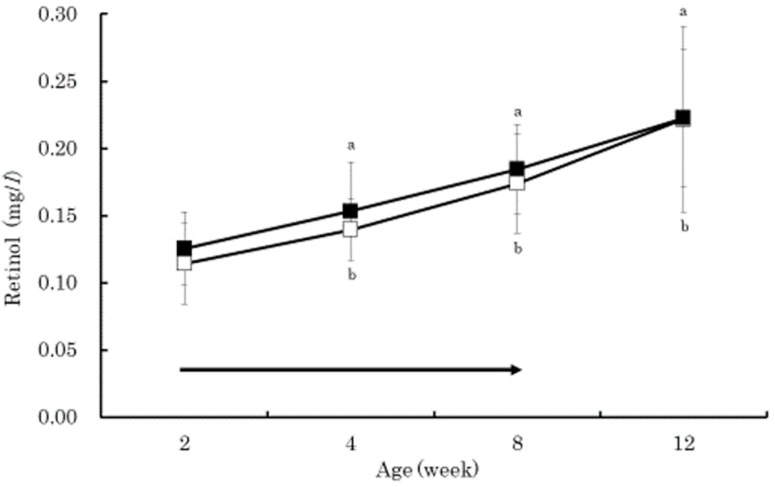
Changes in serum concentrations of retinol in the BC group (dark square) and control group (empty square). Data are shown as mean ± SD. Arrow indicates beta-carotene supplementation period. Same lower case letters indicate a significant difference in concentration when each group is compared to the concentration from 2 weeks of age within each group (*p* < 0.05).

**Figure 3:**
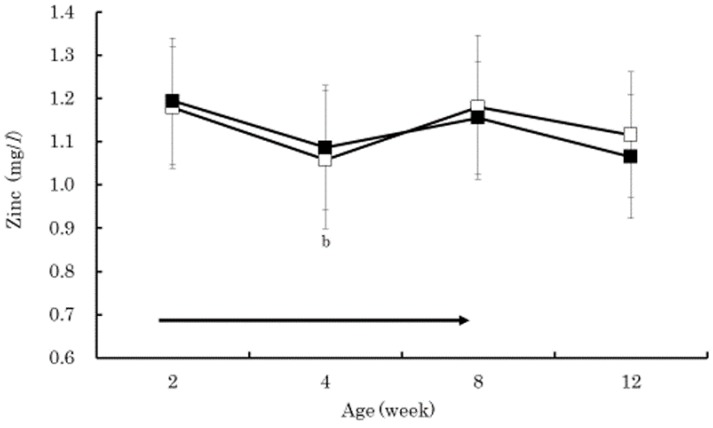
Changes in serum concentrations of zinc in the BC group (dark square) and control group (empty square). Data are shown as mean ± SD. Arrow indicates beta-carotene supplementation period. Lower case letters indicate a significant difference from 2 weeks of age within control group (*p* < 0.05).

**Figure 4:**
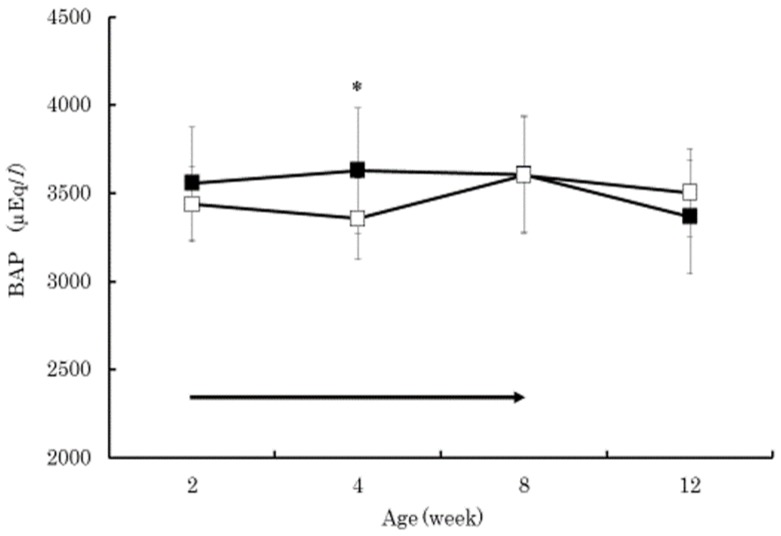
Changes in serum biological antioxidant potential (BAP) in the BC group (dark square) and control group (empty square). Data are shown as mean ± SD. Arrow indicates beta-carotene supplementation period. Asterisks indicate a significant difference between groups at the same age (*: *p* < 0.05).

**Figure 5:**
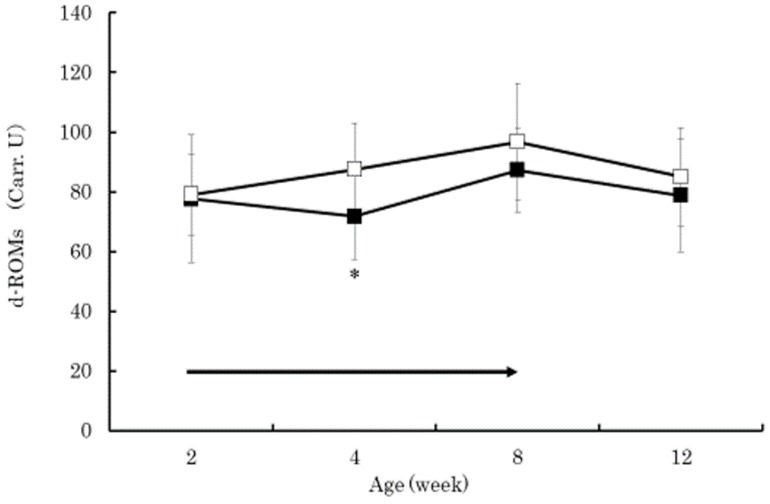
Changes in serum derivatives of reactive oxygen metabolites (d-ROMs) in the BC group (dark square) and control group (empty square). Data are shown as mean ± SD. Arrow indicates beta-carotene supplementation period. Asterisks indicate a significant difference between groups at the same age (*: *p* < 0.05).

**Figure 6:**
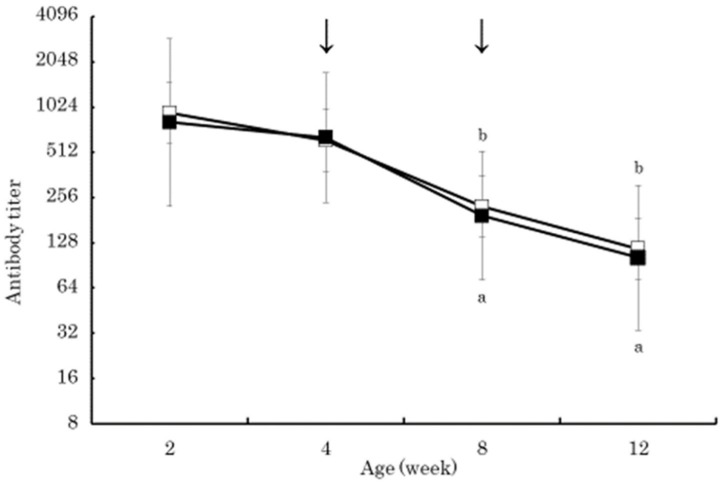
Changes in antibody titers to bovine respiratory syncytial virus in BC group (dark square) and control group (empty square). Antibody titers are shown as mean ± SD. Arrow indicates bovine respiratory syncytial virus vaccination. Same lower case letters indicate a significant difference in concentration when each group is compared to the concentration from 2 weeks of age within each group (*p* < 0.05).

The serum beta-carotene concentrations in the BC group were significantly higher than those in the control group at 4 and 8 weeks of age (*p* < 0.01) (Figure 1). Within the BC group, the serum beta-carotene concentrations at 4 and 8 weeks of age were significantly higher than that at 2 weeks of age (*p* < 0.01). The serum retinol in both groups increased gradually from 2 to 12 weeks of age without significant difference between the groups (Figure 2). The serum retinol concentrations in both groups at 4, 8, and 12 weeks of age were significantly higher than those at 2 weeks of age (*p* < 0.01). The serum zinc concentration in the control group at 4 weeks of age was significantly lower than that at 2 weeks of age (*p* < 0.05) (Figure 3). The serum BAP in the BC group at 4 weeks of age was significantly higher than that in the control group (*p* < 0.05) (Figure 4). The serum d-ROMs in the BC group was lower than that in the control group from 4 to 12 weeks of age (Figure 5), and the difference between groups was statistically significant at 4 weeks of age (*p* < 0.05). The antibody titer in both groups decreased gradually from 2 to 12 weeks of age without significant difference between the groups (Figure 6), and antibody titers in both groups at 8 and 12 weeks of age was significantly lower than those at 2 weeks of age (*p* < 0.01).

## 4. Discussion

Generally, young calves have lower levels of beta-carotene and retinol in the blood due to limited placental transfer [5]. In the present study, the serum retinol concentration in both groups gradually increased and showed similar changes from 2 to 12 weeks of age. However, the serum beta-carotene concentration in the BC group was significantly higher than that in the control group at 4 and 8 weeks of age. These results indicate that supplementing 20 mg/day of beta-carotene in Japanese Black calves is effective at increasing serum beta-carotene concentration with minor influences on serum retinol concentration. Previous studies have also shown that supplementation of beta-carotene to calves did not cause major influences on blood retinol concentration [23,24]. Beta-carotene plays an important role as an antioxidant [4], efficiently contributes to the defense against lipid peroxidation, and scavenges peroxyl radicals [25]. Zinc is also an antioxidant that is similar to beta-carotene [26]. In this study, the serum zinc concentration in the control group, at 4 weeks of age, was significantly lower than the concentration at 2 weeks of age. Therefore, the lower serum BAP concentration in the control group at 4 weeks of age might be due to the decrease in zinc. On the other hand, the serum BAP concentration in the BC group at 4 weeks of age did not decrease, and the serum BAP concentration in the BC group was significantly higher than the control group. Additionally, the serum d-ROMs concentration in the BC group was significantly lower than the concentration in the control group at 4 weeks of age. Increased blood beta-carotene concentration in the BC group caused by supplementation of beta-carotene may have contributed to the increased antioxidative capacity as well as contributing to the reduced production of oxidative stress in blood. Previous studies have also shown that supplementation of beta-carotene to humans decreased several oxidative stress marker concentrations in blood [8,9]. In Buffalo cows, oral administration of commercial supplements containing Aloe arborescence and beta-carotene increased the serum BAP and decreased the serum d-ROMs concentration [7].

Beta-carotene has been shown to have protective effects against infectious diseases by enhancing the immune system [5,6]. Beta-carotene supplementation in humans improved immune function and antibody production in response to vaccination [9]. In cattle, beta-carotene supplementation increased the colostral immunoglobulin concentration in Holstein cows [27]. In this study, despite administration of live bovine RS virus vaccine at 4 and 8 weeks of age, antibody titers gradually declined from 2 to 12 weeks of age in both groups. These changes were thought to be vaccine break due to the influence of the maternal antibody titer. The influence of such maternal antibodies on the response to vaccination in calves has been reported [2,28]. Therefore, it will be necessary to change the timing of live bovine RS virus vaccination.

The present study confirmed that oral beta-carotene supplementation of Japanese Black calves increased blood beta-carotene, reduced oxidative stress, and increased antioxidative capacity. Therefore, oral beta-carotene supplementation to calves is expected to increase resistance to diseases. However, the effect of oral beta-carotene supplementation of Japanese Black calves on the antibody titers in response to live bovine RS virus vaccination was not found to be significant, perhaps due to the influence of the maternal antibody.

In order to enhance health condition in calves, further research is needed to clarify how beta-carotene supplementation reduces oxidative stress and to determine whether oral beta-carotene supplementation is effective for antibody production by vaccination in Japanese Black calves.

## 5. Conclusions

The purpose of this study was to evaluate the effect of beta-carotene supplementation on blood oxidative stress and antibody response to vaccination in calves. Thirty Japanese Black calves were randomly assigned to two groups: 15 calves were orally supplemented with 20 mg/day of beta-carotene from 2 to 8 weeks of age (BC group), and the other 15 calves did not receive supplemented beta-carotene (control group). All calves received commercial modified live bovine RS virus vaccine at 4 and 8 weeks of age. The serum beta-carotene concentrations in the BC group were significantly higher than those in the control group at 4 and 8 weeks of age (*p* < 0.01). The serum retinol in both groups increased gradually from 2 to 12 weeks of age without significant difference between the groups. The serum BAP in the BC group at 4 weeks of age was significantly higher than that in the control group (*p* < 0.05). The serum d-ROMs in the BC group was lower than that in the control group from 4 to 12 weeks of age, and the difference between groups was statistically significant at 4 weeks of age (*p* < 0.05). The antibody titer in both groups decreased gradually from 2 to 12 weeks of age without significant difference between the groups. These results indicated that supplementation of 20 mg/day of beta-carotene in Japanese Black calves was effective at increasing the serum beta-carotene concentration with minor influences on serum retinol concentration, decreasing the serum oxidative stress, and increasing biological antioxidant capacity. However, beta-carotene supplementation did not affect the antibody response to live bovine RS virus vaccination, perhaps due to the presence of the maternal antibody. Further research is needed to clarify how beta-carotene supplementation reduces oxidative stress and if oral beta-carotene supplementation is effective at increasing antibody production when vaccinating Japanese Black calves.

## Figures and Tables

**Table 1:** Amount and nutrient composition of feed without supplement.

Item		Weeks of Age			
		2	4	8	12
Amount (Dry Matter)					
Milk Replacer	(kg)	0.72	0.92	0.92	0.00
Concentrate	(kg)	0.05	0.10	0.50	0.85
Hey (Oats)	(kg)	0.01	0.01	0.05	0.10
Composition (Dry Basis)					
Crude Protein	(%)	30.6	30.1	26.8	18.6
Crude Fat	(%)	15.7	15.2	11.6	2.6
Total Digestible Nutrients	(%)	101.1	100.1	92.9	74.8
Zinc	mg/day	71.8	94.0	124.4	61
Retinol	mg/day	18.9	18.3	14.1	3.5
Beta-Carotene	mg/day	0.0	0.0	0	0.1
